# Therapeutic Hypothermia on Transport: The Quest for Efficiency: Results of a Quality Improvement Project

**DOI:** 10.1097/pq9.0000000000000556

**Published:** 2022-06-14

**Authors:** Stephanie Redpath, Heather Moore, Ewa Sucha, Amisha Agarwal, Nicholas Barrowman, Brigitte Lemyre, Louise St. Germain

**Affiliations:** From the * Department of Pediatrics, Children’s Hospital of Eastern Ontario and University of Ottawa, Ottawa, ON, Canada; † Transport Team, Children’s Hospital of Eastern Ontario, Ottawa, ON, Canada; ‡ Children’s Hospital of Eastern Ontario Research Institute, Ottawa, ON, Canada; § Department of Quality and Patient Safety, The Ottawa Hospital, Ottawa, ON, Canada.

## Abstract

**Introduction:**

Therapeutic hypothermia (TH) within 6 hours after birth is known to improve both survival and neurodevelopmental outcomes in neonates with hypoxic ischemic encephalopathy (HIE). Meeting this recommended target temperature for neonates who require transport for TH treatment can be complex for various reasons. This study aimed to reduce the time from birth to the initiation of TH and target temperature, thereby increasing the proportion of transported neonates reaching target temperature within 6 hours to >50%.

**Methods:**

We evaluated the effect of three quality improvement interventions, including revised transport team processes, outreach education/resources, and the use of a servo-controlled cooling device on land transports. We compared key outcome TH metrics for cohorts before and after implementation.

**Results:**

The study team compared baseline data for 77 to 102 neonates born between 2009 and April 2015 (preintervention) and September 2015 and September 2020 (postintervention(s)). We observed reductions in both the time from birth to the initiation of passive cooling (38%) and time to reach target TH temperature (23%), with an increase in the proportion of neonates reaching target temperature by 6 hours of age from 50% to 71%.

**Conclusions:**

We used quality improvement methodology to identify key areas for intervention(s) and improvement. Targeted interventions have successfully and consistently improved the timing and delivery of TH to neonates with hypoxic ischemic encephalopathy within the transport environment, with a 20% increase in neonates reaching target temperature by 6 hours of age.

## PROBLEM DESCRIPTION

The Children’s Hospital of Eastern Ontario (CHEO) transport team (TT) covers a diverse, predominately rural region of Ontario (440,000 km2). More than 60% of our region’s medical newborn care providers are family medicine, obstetricians, or emergency physicians with limited newborn resuscitation exposure and resources.^1^ In 2015, we completed a retrospective review of CHEO’s therapeutic hypothermia (TH) regional transport practices that identified several clinical care challenges and delays in initiating passive cooling and reaching the target TH temperature within the recommended 6-hour window.^2^ Due to an expansive geographical region, with both access and resource challenges, we found waiting for the TT to arrive at the referral hospital before the initiation of TH resulted in significant delays, especially for neonates born in centers outside the urban area.

## AVAILABLE KNOWLEDGE AND RATIONALE

Hypoxic ischemic encephalopathy (HIE) from perinatal asphyxia/birth depression occurs in 1–2 per 1,000 live births.^3^ There is evidence that induced hypothermia (33–34 °C) within 6 hours of birth significantly improves survival and neurodevelopmental outcomes in neonates with moderate to severe HIE.^4,5^ Therefore, TH is now considered standard of care treatment provided within tertiary neonatal intensive care (NICU) facilities.^4,6^ Unfortunately, many neonates with HIE who qualify for TH are born in hospitals that do not offer this therapy, necessitating clinical stabilization followed by transport to our regional tertiary center for treatment. In the referral hospital and transport environment, passive hypothermia is initiated until servo-controlled active cooling can be applied at the tertiary center. However, the availability of portable servo-controlled cooling devices (SCDs) for transport makes it possible for teams to commence active cooling at the referring unit upon their arrival, decreasing the time to target temperature range with enhanced temperature control management compared to passive cooling.^7–13^ Despite the demonstrated benefits of SCD’s when used in transport,^10,12^ more recent studies continue to show achieving target temperature within the 6-hour window remains a challenge for TTs with proportions between 55% and 63%.^8^ Given both the timing and safe delivery of TH are imperative to optimize patient outcome(s),^13,14^ early identification and referral of patients for tertiary-level clinical support are essential, with TH initiation pending the arrival of TTs.^15,16^

## SPECIFIC AIMS

We conducted the quality improvement (QI) study that described to improve the effectiveness of our TH treatment within the transport environment. Through specific clinical process changes and consistency in management, we sought to reduce both the time from birth to the initiation of TH and target temperature, thereby increasing the proportion of transported neonates reaching target temperature within 6 hours to greater than 50%. This report outlines the three QI interventions initiated and correlates these key outcome measures.

## METHODS

### Context

Transferring a neonate with suspected HIE first involves physicians in regional centers calling our tertiary care NICU for clinical advice and activating transport. Then, a neonatologist and TT members provide the consultation. As described in the National Institute of Child Health and Human Development (NICHD) study,^16^ neonates who meet the criteria can receive TH. Advice regarding the initiation of TH using passive cooling (discontinuation of radiant heat and unbundling while closely monitoring peripheral temperatures) or awaiting further evaluation by the TT is based upon the clinical scenario, findings on examination, availability of the team, and distance to travel. If passive cooling fails to lower core temperature, the TT will use gel packs to actively cool patients,^17,18^ with continuous rectal temperature monitoring to maintain a core temperature between 33 °C and 34 °C. If a patient’s temperature falls below a threshold, the TT will actively implement maneuvers to warm the patient. Since June 2017, the SCD has been providing active cooling throughout all land transports. Within Canada, medical air transport is provided in partnership with provincial air transport resources. At this time, SCDs are not certified by Transport Canada for use on any aircraft.

### Improvement Interventions

**Fig. 1.**
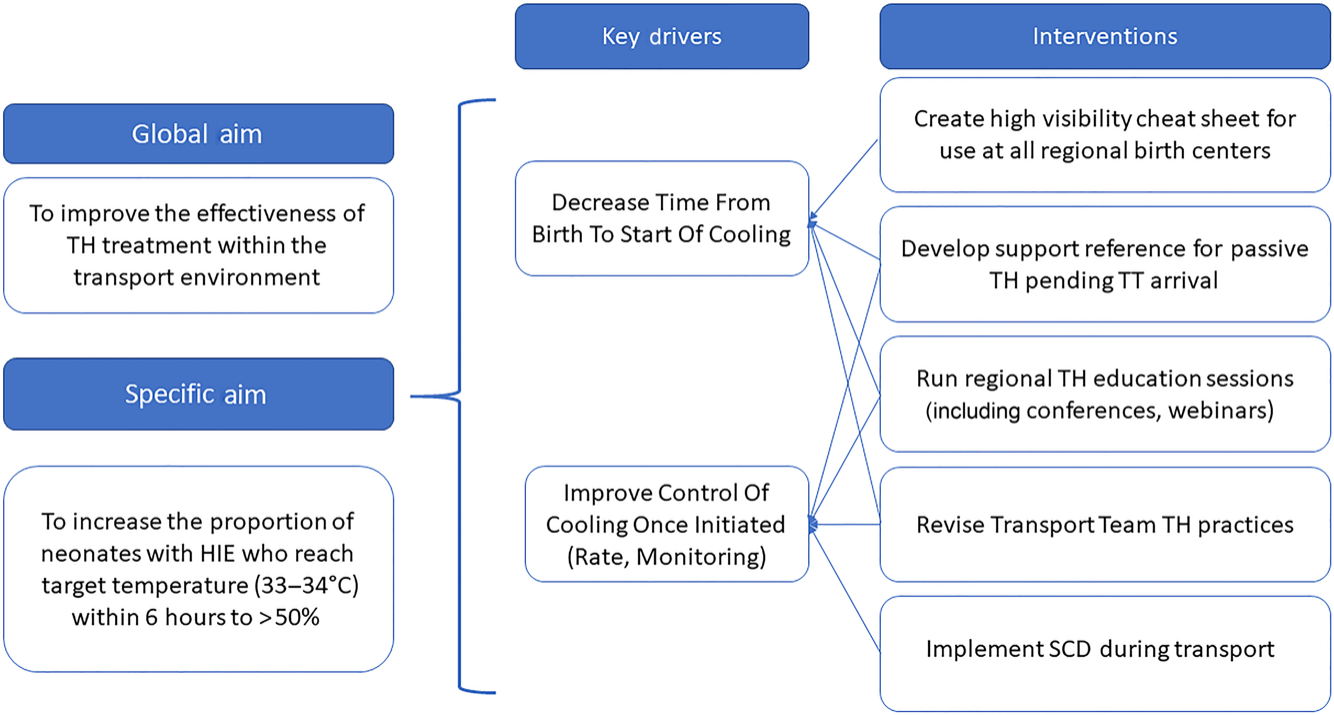
Key driver diagram.

Following our retrospective study in 2015,^2^ QI methodology was used to identify key areas for intervention(s) and improvement. We mapped the existing processes for identifying and managing neonates committed to TH and requiring transport, considering each step carefully. Given the number of Neonatologists providing consultation to referral physicians, we recognized the need for consistency in the passive cooling initiation process and TT TH clinical care practices. We also identified the need to develop targeted outreach regional education resources, specifically considering regional resource variations and their reported limitations. Our key driver diagram demonstrates the relationship between our study’s aim(s), with primary drivers and interventions contributing to achieving the aim (Fig. 1).

**Fig. 2.**
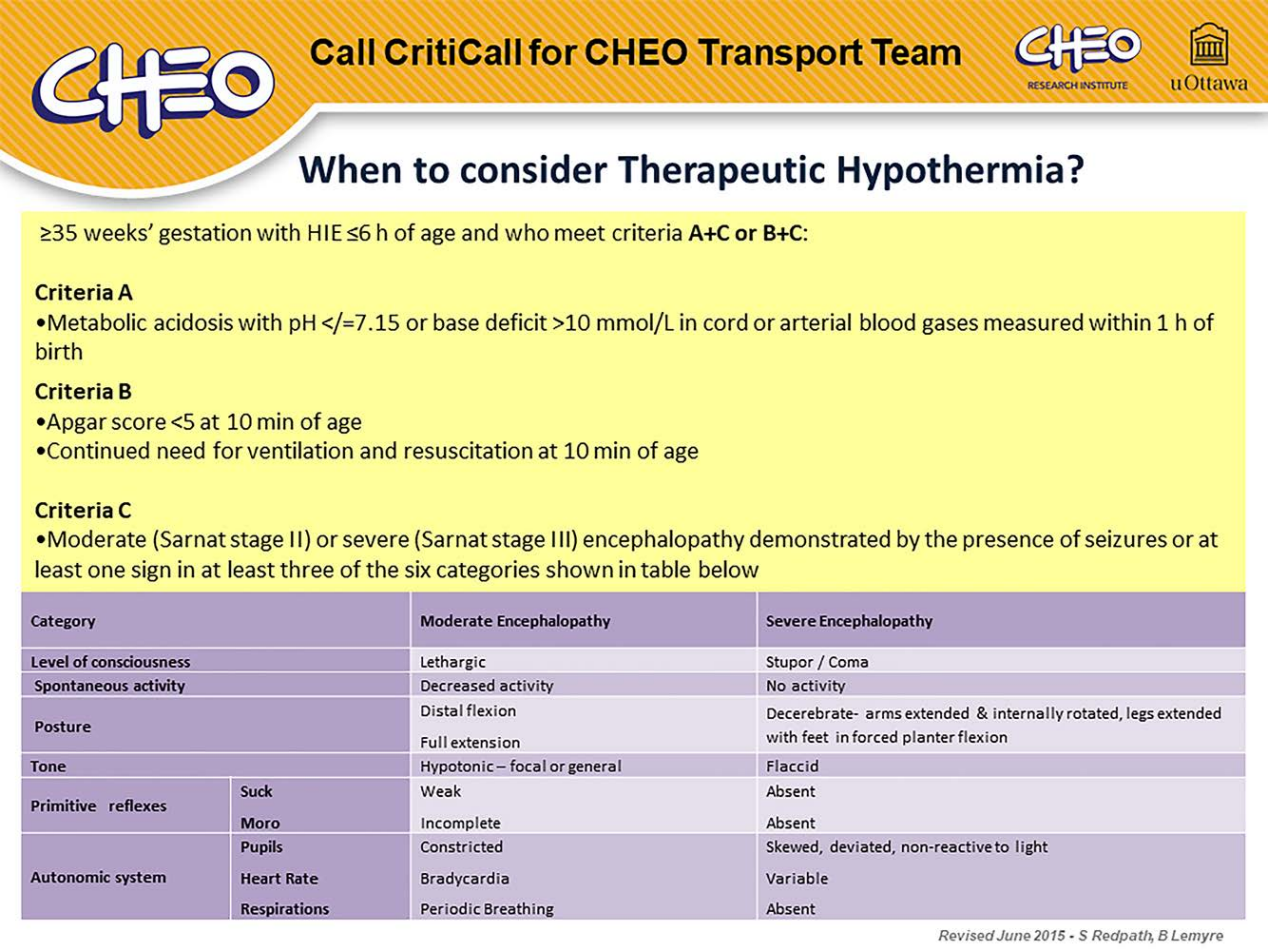
“When to Call” template.

Measures implemented between April and September 2015 included:

- Outreach resources: a high visibility cheat sheet was created for use in all birthing and nursery facilities, emphasizing key criteria for early consideration of patient suitability for TH, including the direct tertiary-level number for on-call support information (Fig. 2). Given the majority of our regional referral centers do not have continuous rectal temperature monitoring experience or capabilities, we also created a passive hypothermia guideline with an adaptation, as per Sussman and Weiss,^19^ allowing for axillary temperature monitoring pending arrival of the TT. This guideline is faxed (or emailed) to the regional referral center as a support reference to promote safe and effective early initiation of TH using passive cooling when advised by a neonatologist (**see Appendix 1, Supplemental Digital Content 1,** http://links.lww.com/PQ9/A373). We disseminated these resources through teaching within local hospital TH education sessions, regional conferences, webinars and uploaded to the CHEO TT website.
- TT practice change: we established a more rigorous and accountable process that requires standardized neurological examination immediately on the team’s arrival with vigilant documentation and temperature management throughout the transport (**see Appendix 2, Supplemental Digital Content 2,** http://links.lww.com/PQ9/A373).

Measure implemented between June 2017 and June 2020 included:

- A portable SCD (Tecotherm Neo, Inspiration Healthcare, International Biomedical, Austin, Tex.) was introduced on all land transports.

### Measures

This single-center QI study included all neonates committed to TH and transported by the regional neonatal TT to CHEO, Ottawa, ON, Canada, between October 2009 and September 2020. We used QI methods and run charts to evaluate the interventions implemented between April and September 2015 and added the SCD in June 2017. The primary outcome measures for our improvement work were the percentage of neonates who achieved target temperature by 6 hours and the related measure of time from birth to target temperature in neonates with HIE requiring transport to a tertiary care center. The key process measure was the time from birth to the initiation of TH. We compared baseline data and TH in transport metrics (process measures) for neonates born between October 2009 and April 2015 (preintervention cohort) to those born between September 2015 and September 2020 (postintervention(s)). We described the demographic and clinical characteristics of the neonates in the preintervention and postintervention cohorts using means, medians, and proportions. Median transport times were summarized using medians and interquartile range (IQR) and compared using a two-sample Wilcoxon test with a *P* value of 0.05.

### Analysis

Data analysis was completed using RStudio version 1.3.1093 (RStudio Team [2020]. RStudio: Integrated Development Environment for R. RStudio, PBC, Boston, Mass., http://www.rstudio.com/). We used run charts for our measures of time from birth to the initiation of TH and target temperature, considering established rules to help determine whether or not the variation within the dataset is due to the random variation typical of the performance of that process or due to nonrandom attributable change in the process.^20,21^ The Wilson-type p chart was used to outline the percentage of neonates who achieved target temperature by 6 hours, calculate the upper and lower limits, and account for a small sample size each year.^22^

### Ethical Considerations

This study underwent a formal ethics review and approval by CHEO’s Research Ethics Board Committee (Study reference #20190373).

## RESULTS

### Demographics

Within the outlined time frame, 195 neonates exist in the CHEO TH dataset. Although this study focuses on our center and TT approach, we excluded 16 neonates (n = 16) transported by another team and/or had missing data. Therefore, the dataset included 179 neonates committed to TH and transported by the regional TT between October 2009 and September 2020. Baseline characteristics are similar between the neonates preintervention(s) and postintervention(s) (Table 1).

**Table 1.** Descriptive Statistics of Cohorts (N = 179)

Characteristic	Preintervention, N (Missing Data)	Preintervention, N (%)/Mean (SD)	Postintervention, N (Missing Data)	Postintervention, N(%)/Mean (SD)
Birthweight (g)	77 (0)	3,311.8 (582.6)	102 (0)	3,350.0 (665.4)
Gestational age (d)	77 (0)	275.3 (11.9)	102 (0)	273.2 (12.5)
Sex	77 (0)		102 (0)	
Female		34 (44.2)		37 (36.3)
Male		43 (55.8)		65 (63.7)
In-town vs out-of-town birth	77 (0)			
In-town		59 (76.6)		68 (66.7)
Out-of-town		18 (23.4)		34 (33.3)
Birth hospital (level of NICU)	77 (0)		102 (0)	
Level 3		21 (27)		16 (16)
Level 2		44 (57)		65 (64)
Level 1		12 (16)		21 (20)
Apgar score 1 min	77 (2)	1.1 (1.1)	102 (2)	1.5 (1.5)
Apgar score 5 min	77 (1)	2.6 (1.7)	102 (2)	3.2 (2.0)
Apgar score 10 min	77 (74)	5.3 (1.5)	102 (11)	4.4 (2.0)
Cord pH	77 (18)	6.9 (0.2)	102 (16)	7.0 (0.2)
Cord BE	77 (74)	−20.6 (2.4)	102 (28)	−12.6 (6.7)
First gas pH	77 (3)	7.0 (0.2)	102 (4)	7.1 (0.2)
Patient temperature on arrival of TT to referral Hospital (°C)	77 (2)	35.5 (1.3)	102 (0)	35.3 (1.3)
Patient temperature on arrival to tertiary hospital (°C)	77 (2)	34.0 (1.2)	102 (1)	34.4 (1.2)
Recommended passive cooling	77 (2)	44 (58.7)	102 (0)	83 (81.4)

One hundred ninety-five infants exist in the transport TH dataset within the outlined time frame—16 patients excluded as either transported by another team or had missing data.

### Key Outcome and Process Measures

**Fig. 3.**
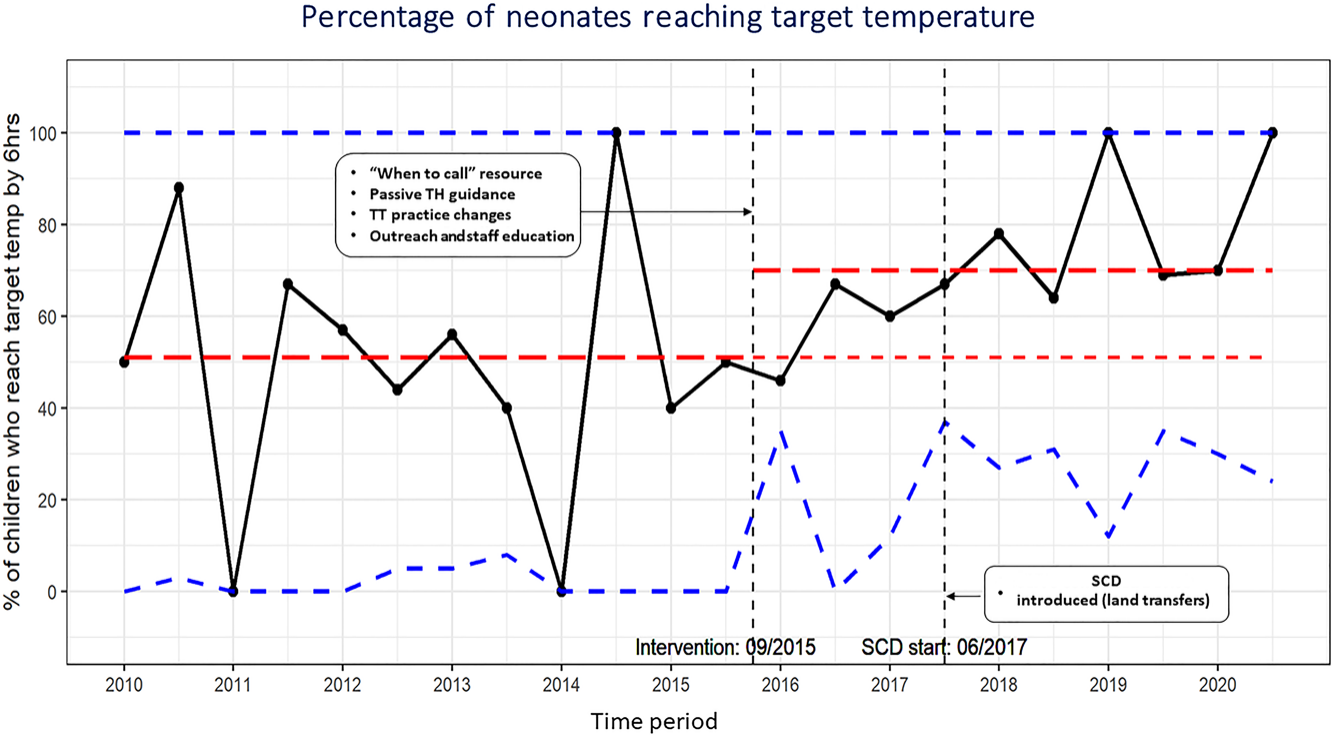
Percentage of neonates reaching target temperature. We used a Wilson-type p chart to calculate the upper and lower limits and account for a small sample size in each year.^22^ Black line is the proportion of children that achieved target temperature by 6 hours. Red line is the average proportion of children that achieved target temperature by 6 hours over each time period (preintervention and postintervention). Blue line is the lower and upper control limits for the process calculated using three SDs from the mean. k = 3 (according to the American Standard and based on control limits with the target false alarm rate of 0.0027. The values of upper and lower limits were capped at 0% and 100%, respectively, since proportions below 0% and above 100% do not make sense.

Comparing key outcome and process measures preintervention/postintervention (Table 2), we found a reduction in both the median time from birth to the initiation of cooling by 38% to 1.8 hours (IQR 0.9, 3.4) and birth to the target temperature by 23% to 4.6 hours (3.1, 6.4). The percentage of neonates for whom passive cooling is initiated at the referral center (pending arrival of the TT) increased from 59% to 81%. The number of transported neonates within the target temperature range by 6 hours of age increased from 50% to 71% (*P* = 0.01) (Fig. 3). There was no appreciable difference in the timing of birth to referral, referral to the arrival of the TT, or initiation of cooling to the target temperature without using the SCD. The number of neonates with a temperature less than 33 °C on the arrival of the TT to the referral center was unchanged [n (%)]: 4 (5.3%) and 5 (4.9%) for preintervention and postintervention periods, respectively.

**Table 2.** TH on Transport Variables, Preintervention versus postintervention (N = 179) Values <0.05, Marked with Asterisks, Are Considered Statistically Significant

Transport TH variables (h)	Pre		Post		Wilcoxon Test
	N (missing)	Median (Q1, Q3)	N (missing)	Median (Q1, Q3)	*P*
Birth to initiation of cooling	77 (8)	2.9 (1.4, 4.4)	102 (3)	1.8 (0.9, 3.4)	0.01*
Birth to target temperature	77 (5)	6.0 (4.0, 8.2)	102 (6)	4.6 (3.1, 6.4)	0.025*
Birth to referral	77 (3)	1.5 (0.9, 2.0)	102 (45)	1.3 (0.7, 2.1)	0.434
Referral to initiation of cooling	77 (7)	1.1 (0.0, 2.4)	102 (3)	0.3 (-0.2, 1.1)	0.012*
Referral to arrival of TT	77 (3)	1.1 (0.8, 1.9)	102 (0)	1.0 (0.8, 2.1)	0.533
Referral to target temperature	77 (5)	3.9 (2.7, 5.5)	102 (6)	3.0 (2.0, 5.4)	0.048*
Initiation of cooling to target temperature	77 (10)	2.7 (1.4, 3.9)	102 (9)	2.3 (1.2, 3.4)	0.261

Considering the geographical influence on the timing of arrival of the TT, including inclement weather and/or air transport requirements, we specifically reviewed out-of-town neonates (defined as referrals from >20 km from the tertiary TH center) as a subgroup for analysis (**see Appendix 3, Supplemental Digital Content 3,** http://links.lww.com/PQ9/A373). Among our out-of-town neonates (N = 52), we observed substantial reductions in the median time from referral to the initiation of passive cooling from 3.1 (2.2, 4.4) to 0.5 hours (0.2, 2.2), birth to the initiation of cooling from 4.1 (3.1, 5.5) to 2 hours (1.3, 3.8), and time to target temperature from 8.2 (5.3, 9.2) to 5.4 hours (3.4, 7.3).

Since the introduction of the SCD on land transports in June 2017 (**see Appendix 4, Supplemental Digital Content 4,** http://links.lww.com/PQ9/A373), we observed further reductions in the time taken to reach the set target temperature from 5.9 (4.5, 8.4) to 3.6 hours (2.7, 4.6), *P* < 0.001, and the median time from the initiation of cooling to target temperature decreased from 3.1 (1.3, 4.2) to 2 hours (1.3, 2.5), *P* = 0.057, with no reported adverse events.

**Fig. 4.**
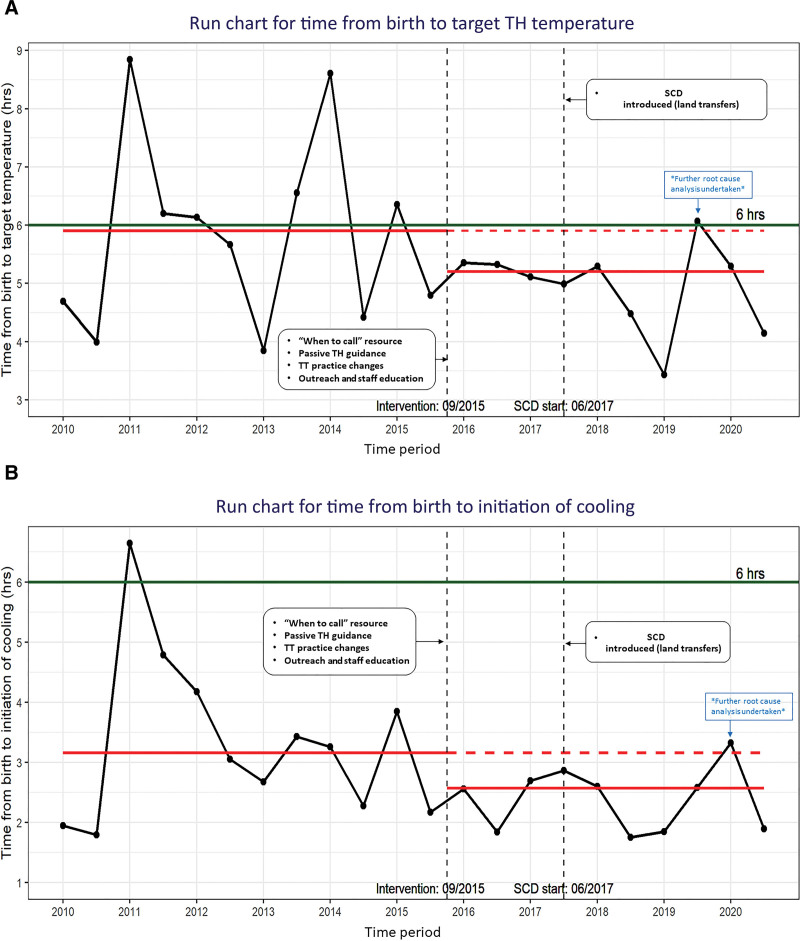
Run control charts that demonstrate the trends between 2010 and 2020, grouped biannually. A, Run chart for the median time from birth to target temperature. B, Run chart for the median time from birth to initiation of cooling. Each point on the chart represents the median value of time from birth to target temperature (A) and birth to initiation of cooling (B) biannually. CL is the median of time from birth to target temperature over the years and represents the process center. CL was calculated separately for the preintervention and postintervention periods. The green line represents the time from birth to target temperature equal to 6 hours. The year 2009 was excluded from the chart as only two measurements were available.

Figure 4A, B is run control charts that demonstrate the trends between 2010 and 2020, grouped biannually, for the median time from birth to target temperature and the median time from birth to the initiation of cooling. For both measures, lower (shorter) times are favorable. Both control charts demonstrate an encouraging trend towards improvement with six or more successive points below the baseline median during the postintervention period, suggesting the observed improvements resulted from the interventions rather than natural random variation.

## DISCUSSION

Although the literature recommends that the timing and safe delivery of TH are crucial to optimize outcome(s) for neonates with HIE,^4,5,13,14^ meeting these requirements in neonates who need transport for treatment is especially challenging within a broad geographical region with variable resources and clinical expertise. Through the introduction of targeted TT practice changes and specifically adapted outreach support measures, we have successfully increased the number of neonates for whom passive cooling is being initiated within the referral center and decreased the time taken to reach the intended temperature in a higher percentage of our neonates, without an increase in adverse events.

An area identified in our first review of practice that required substantial improvement was the initiation of passive cooling in neonates qualifying for TH.^2^ Delays identified were found to be the cumulative result of varying regional hospital clinical expertise and resources, a limited ability to monitor rectal or esophageal temperatures appropriately,^4,6^ distances involved, and our neonatologists perceived risk of overcooling. Roberts et al.^23^ describe similar clinical care challenges in Australia based on vast geography and referral hospital capabilities. Initiating passive cooling at referral centers before the transfer is essential to achieve target core temperature faster.^15,16,23^

Although correlated, axillary temperatures in newborns do not accurately reflect rectal or core temperatures based on systematic reviews,^24^ precluding the use of axillary monitoring as a proxy for rectal temperature measurement in TH protocols.^6,25^ Unfortunately, most of our regional referral centers have neither rectal temperature monitoring experience nor resource capabilities. Sussman and Weiss^19^ recommended that centers not accustomed to caring for critically ill newborns consider maintaining the axillary temperature between 33.5 °C and 35 °C as an alternative to avoid unnecessary injury from incorrect techniques. Faced with this limitation within our region and aware of the need to consider adapting to meet the ongoing challenge of highly variable community resources,^1^ we created a passive cooling guideline (**see Appendix 1, Supplemental Digital Content 1,** http://links.lww.com/PQ9/A373). This guideline allows for the use of axillary temperature monitoring as described, minimizing the risk of injury and/or systemic adverse effects, nevertheless providing a degree of neuroprotection until the TT arrives. This considered approach has enabled our community partners to successfully initiate passive cooling within the referral center pending the arrival of the TT without an increase in patients being overcooled or documented adverse events. The success of this approach is most striking in the out-of-town patient subgroup. Given TT access to this patient subgroup is frequently challenged by complex transport logistics and weather extremes, ensuring the appropriate care practices be performed safely and promptly is fundamental in our efforts to optimize care across the region.

Using an SCD for cooling on transport has consistently demonstrated a shorter time to target temperature with more predictable temperature regulation.^7–13^ Akula et al.^10^ highlighted the importance of this equipment for out-born neonates in regions with prolonged transport times due to large geographical areas and/or delays in treatment initiation. Since the introduction and use of the SCD for all land transports, our results are in keeping with these findings. Figure 3 also illustrates a further increase in the percentage of neonates reaching target temperature on admission to CHEO beyond June 2017. Unfortunately, we cannot use this equipment for all TH cases in our region because of restrictions precluding its use in aircraft within Canada. We, therefore, emphasize the importance of ongoing targeted regional measures, outreach education, and advocacy for SCD certification, allowing for its use in medical air transport.

### Strengths and Limitations

Our project has several strengths. A multidisciplinary approach, high compliance rate despite the challenging widespread geographical conditions and resource variation(s), and an improved outcome when offering TH to our neonatal population affected by HIE. This result highlights that center-specific transport guidelines (targeted measures) can be successfully implemented. We acknowledge the following limitations: this study is a QI initiative in a single-center institution without formal evaluation of the different modes of cooling during transport, specifically given standardized recommendations and studies that already exist in this domain.^7–10,12,17,19^ Although we collected developmental outcome data on all our TH patients, we did not link cases to developmental outcomes for this study. The earlier neonates eligible for TH have passive cooling safely initiated, the lower the risk of missing an opportunity to reach better outcomes.^4,5,14^ The run charts in Figure 4A, B demonstrate an encouraging trend toward improvement. However, we acknowledge that Shewhart (control) charts are required to determine the stability of this new process and whether the improvements are sustainable.^26^ The low frequency of our TH in transport cases per year impacts the data collection rate and the time frame required to fully satisfy accepted ASQ rules for identifying special cause variation in a control chart.^27^ Considering this study is part of our continuous QI practice in transport, analysis of the process stability, and shift from the baseline using a control chart will be included in the study’s ongoing process monitoring. For example, having observed a slight increase in the target temp in 2019 on our run chart, the QI team formally reviewed all 2019 TH cases. We identified some inconsistencies in-process and a need for neonatologist feedback and ongoing TT education. In the meantime, the centerline shift in data identified on the run chart was both encouraging and meaningful to our study team, which we felt may be important for replicability elsewhere and/or impact a similar system.

## CONCLUSIONS

This QI study outlines our practical and implementation experience demonstrating the need for various clinical care strategies, adaptations, and targeted measures to optimize TH’s timing and safe delivery to neonates with HIE within the prehospital and transport environment. The time-sensitivity of patient recognition, treatment requirements, and the realities of long-distance transport emphasize the need for timely transport access and specialized equipment to promote system improvement across all domains in the circle of care.

## DISCLOSURE

The authors have no financial interest to declare in relation to the content of this article.

## ACKNOWLEDGMENTS

The authors would like to thank all neonatal TT members who cared for our patients while obtaining and recording the data upon which this study is based and Prof. Emanuela Ferretti for critical review of the manuscript. We would also like to thank the neonates and their families.

## Supplementary Material


