# Supplementary material for: Therapeutic Hypothermia on Transport: The Quest for Efficiency: Results of a Quality Improvement Project
Source: Pediatr Qual Saf. 2022 Jun 14;7(3):e556. doi: 10.1097/pq9.0000000000000556 (PMC9197372; doi:10.1097/pq9.0000000000000556)
Supplement: Supplementary file 1 [file pqs-7-e556-s001.pdf]

PASSIVE HYPOTHERMIA GUIDELINE

Recommendation for the initiation of passive cooling pending Transport Team arrival:

1. Turn off radiant warmer or decrease isolette temperature to 24-26ºC

2. Clearly document time of initiation

3. Aim for target temperature of 33-34ºC

4. Rectal temperature monitoring recommended *
*Centers not accustomed to caring for critically ill newborns or do not frequently use rectal temperature measurements may consider maintaining the axillary temperature between 33.5-35ºC

5. Check rectal (or axillary) temperature every 15 minutes

6. If infant temperature decreases below 33ºC (33.5ºC axillary) turn on radiant warmer or isolette with the temperature set 0.5ºC above the infant's temperature

7. Do not use/add ice packs or cold packs

Sussman et al. Adv Neonatal Care. 2013

Cooling Guidelines for Neonatal Transport

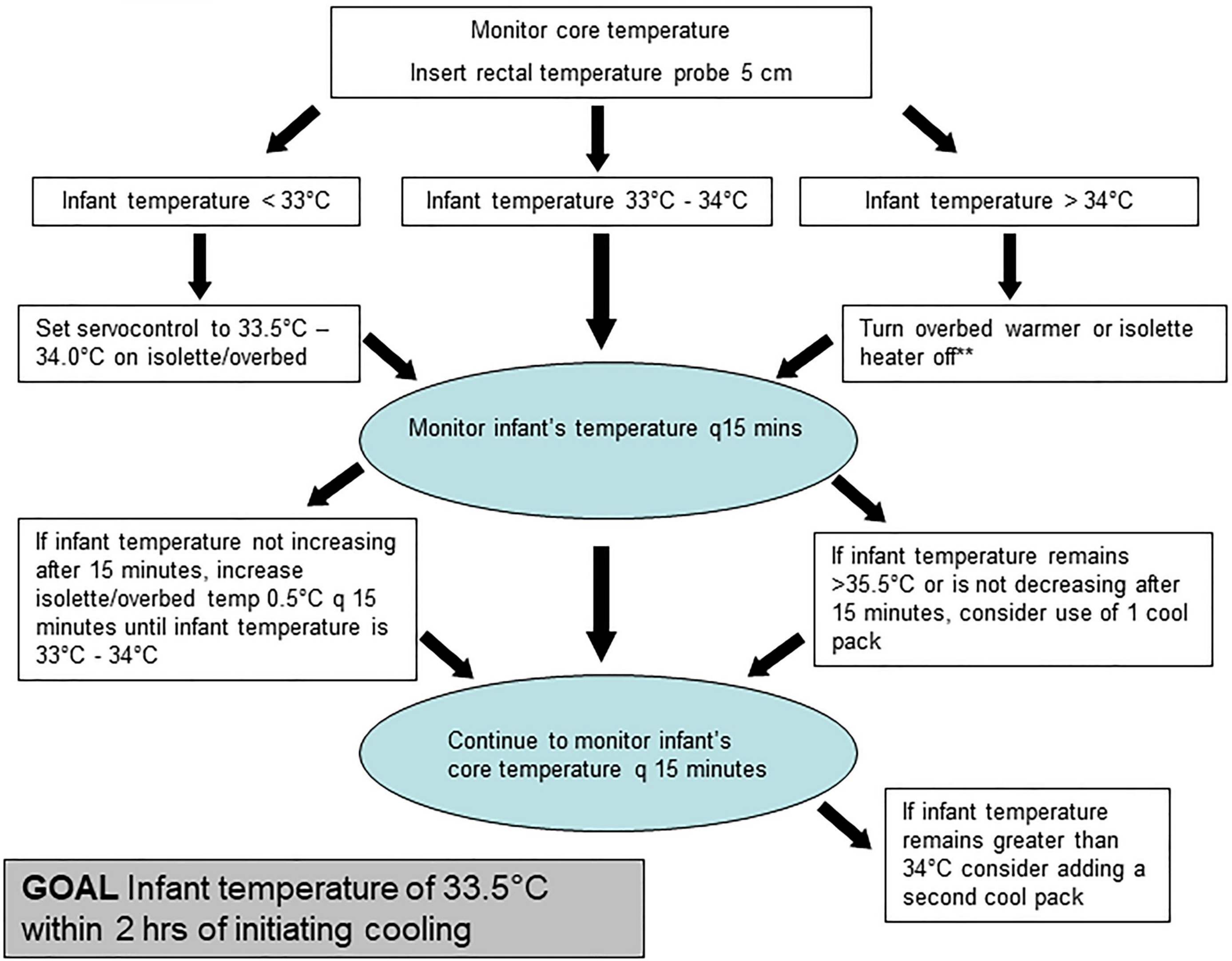

**If patient not ventilated, require open isolette port hole for air flow

NNTT Arrival Time ______
Rectal Probe Insertion Time ______
Initial Rectal Temperature ______

Time of Passive Cooling Start ______
Time of Active Cooling Start ______
Time ≤ 34°C Reached ______

GOAL Infant temperature of 33.5°C within 2 hrs of initiating cooling

Data from Jacobs et al [2], Kendall et al [9]

Table 3: TH on Transport variables - Out-of- town patients (N=52), pre versus post-intervention

Out-of-town patients - defined as referrals from >20km from the tertiary TH center

**Transport TH variables (hrs.)**	**Pre (n=18)**	**Post (n=34)**	**Wilcoxon Test**
	Median (Q1,Q3)	Median (Q1, Q3)	p-value
Birth to initiation of cooling	4.1 (3.1, 5.5)	2.0 (1.3, 3.8)	0.017*
Birth to target temperature	8.2 (5.3, 9.2)	5.4 (3.4, 7.3)	0.026*
Birth to referral	1.0 (0.7, 1.5)	1.1 (0.6, 1.6)	0.825
Referral to initiation of cooling	3.1 (2.2, 4.4)	0.5 (0.2, 2.2)	0.03*
Referral to arrival of NNTT	2.7 (1.9, 5.3)	2.5 (1.7, 4.1)	0.387
Referral to target temperature	6.7 (4.0, 7.5)	3.7 (2.5, 5.4)	0.018*
Initiation of cooling to target temperature	2.6 (1.8, 4.2)	2.0 (1.1, 3.4)	0.163

Table 4: TH on Transport - SCD used vs not used, 2017 to 2020, Land transport only (N=76)

values < 0.05, marked with asterisks, are considered statistically significant.

**Transport TH variables (hrs.)**	**SCD No**		**SCD Yes**		**Wilcoxon Test**
	N (missing)	Median (Q1, Q3)	N (missing)	Median (Q1,Q3)	p-value
Birth to initiation of cooling	31 (0)	2.4 (0.9, 5.5)	45 (3)	1.4 (0.9, 2.5)	0.059
Birth to target temperature	31 (2)	5.9 (4.5, 8.4)	45 (0)	3.6 (2.7, 4.6)	<.001*
Birth to referral	31 (0)	1.2 (0.8, 2)	45 (0)	1.2 (0.6, 2.1)	0.788
Referral to initiation of cooling	45 (3)	0.3 (-0.3, 0.8)	31 (0)	0.4 (0.0, 4.2)	0.062
Referral to arrival of NNTT	31 (0)	1 (0.8, 1.6)	45 (0)	1 (0.9, 1.8)	0.529
Referral to target temperature	31 (2)	5.4 (3.3, 6.9)	45 (0)	2.5 (1.5, 3.1)	<.001*
Initiation of cooling to target temperature	31 (2)	3.1 (1.3, 4.2)	45 (3)	2 (1.3, 2.5)	0.057
